# L-Cysteine Administration Attenuates Pancreatic Fibrosis Induced by TNBS in Rats by Inhibiting the Activation of Pancreatic Stellate Cell

**DOI:** 10.1371/journal.pone.0031807

**Published:** 2012-02-16

**Authors:** LiJuan Yang, JiaQing Shen, ShanShan He, GuoYong Hu, Jie Shen, Feng Wang, Ling Xu, WeiQi Dai, Jie Xiong, JianBo Ni, ChuanYong Guo, Rong Wan, XingPeng Wang

**Affiliations:** 1 Department of Gastroenterology, The First People's Hospital, Shanghai Jiao Tong University School of Medicine, Shanghai, People's Republic of China; 2 Department of Gastroenterology, The Tenth People's Hospital of Shanghai, Tongji University, Shanghai, People's Republic of China; Technische Universität München, Germany

## Abstract

**Background and Aims:**

Recent studies have shown that activated pancreatic stellate cells (PSCs) play a major role in pancreatic fibrogenesis. We aimed to study the effect of L-cysteine administration on fibrosis in chronic pancreatitis (CP) induced by trinitrobenzene sulfonic acid (TNBS) in rats and on the function of cultured PSCs.

**Methods:**

CP was induced by TNBS infusion into rat pancreatic ducts. L-cysteine was administrated for the duration of the experiment. Histological analysis and the contents of hydroxyproline were used to evaluate pancreatic damage and fibrosis. Immunohistochemical analysis of α-SMA in the pancreas was performed to detect the activation of PSCs in vivo. The collagen deposition related proteins and cytokines were determined by western blot analysis. DNA synthesis of cultured PSCs was evaluated by BrdU incorporation. We also evaluated the effect of L-cysteine on the cell cycle and cell activation by flow cytometry and immunocytochemistry. The expression of PDGFRβ, TGFβRII, collagen 1α1 and α-SMA of PSCs treated with different concentrations of L-cysteine was determined by western blot. Parameters of oxidant stress were evaluated in vitro and in vivo. Nrf2, NQO1, HO-1, IL-1β expression were evaluated in pancreas tissues by qRT-PCR.

**Results:**

The inhibition of pancreatic fibrosis by L-cysteine was confirmed by histological observation and hydroxyproline assay. α-SMA, TIMP1, IL-1β and TGF-β1 production decreased compared with the untreated group along with an increase in MMP2 production. L-cysteine suppressed the proliferation and extracellular matrix production of PSCs through down-regulating of PDGFRβ and TGFβRII. Concentrations of MDA+4-HNE were decreased by L-cysteine administration along with an increase in GSH levels both in tissues and cells. In addition, L-cysteine increased the mRNA expression of Nrf2, NQO1 and HO-1 and reduced the expression of IL-1β in L-cysteine treated group when compared with control group.

**Conclusion:**

L-cysteine treatment attenuated pancreatic fibrosis in chronic pancreatitis in rats.

## Introduction

Chronic pancreatitis (CP) is characterized by progressive fibrosis and pain and/or loss of exocrine and endocrine functions [1], [2]. Conservative modalities include analgesics, anti-secretory therapy with H_2_ receptor blockers or proton pump inhibitors and pancreatic enzyme supplementation. However, clinical studies including randomized control trials have shown divergent results questioning the efficacy of these modalities [3]. Also, on the interventional front (endotherapy [4], celiac plexus neurolysis and block [5], and surgery [6]), there is a paucity of convincing efficacy data. Studies have also shown that high-dose naproxen, which is orally used for the treatment of pain, can aggravate pancreatic fibrosis in a rat model of chronic pancreatitis [7].

Currently, numerous *in vivo* and *in vitro* studies have provided strong evidence for a pivotal role for pancreatic stellate cells (PSCs) in fibrogenesis associated with acute and chronic pancreatitis [8]–[11]. In addition to the elimination of the conditions inducing acinar cell injury (e.g., alcohol) and the reduction of the inflammatory response of the host, therapeutic targeting of PSCs may represent a promising new strategy for reducing fibrogenesis. Pharmacological agents have been developed to inhibit the activation and functions of PSCs such as anti-inflammatory and immunomodulatory compounds, antioxidant compounds, protease inhibitors and the HMG-CoA reductase inhibitor [12].

Oxidative stress is an important stimulus of PSCs activation [13]. In cell culture experiments, it has been shown that rat PSCs are activated in response to ethanol *per se*, mediated by the generation of oxidant stress. Exposure to a pro-oxidant complex, such as iron sulphate/ascorbic acid (which increases oxidant stress within PSCs) leads to PSCs' activation [14]. Therefore, antioxidant activity is effective for anti-fibrogenesis.

L-cysteine, the limiting amino acid for glutathione (GSH) synthesis [15], is a sulfur-containing amino acid and plays an important role as an extracellular reducing agent. *In vitro* studies have shown that antioxidants, such as N-acetylcysteine and vitamin E, can prevent oxidant stress or ethanol-induced PSCs activation [16], [17]. We hypothesize that L-cysteine, as an anti-oxidant compound, may prevent pancreatic fibrosis and inhibit PSCs activation. We therefore evaluated the effect of L-cysteine *in vivo* and *in vitro*, determining whether it was effective in preventing the development of pancreatic fibrosis induced by trinitrobenzene sulfonic acid (TNBS) in a rat model *via* its effect on PSCs. Here we report the antifibrotic effect of L-cysteine in chronic pancreatitis induced by TNBS administration in rats.

## Materials and Methods

### Ethics statement

All the animal related procedures were approved by the Animal Care and Use Committee of The Tenth People's Hospital of Shanghai. Permit number: 2011-RES1. This study was also approved by Science and Technology Commission of Shanghai Municipality (ID: SYXK 2007-0006). The rats were kept at 18°C–26°C on a 12 hours light and dark cycle with free access to water and standard rat chow. They were allowed to acclimatize for a minimum of 1 week. The environment was maintained at a relative humidity of 30%–70%.

### Animals and experimental protocol

**Figure 1:**
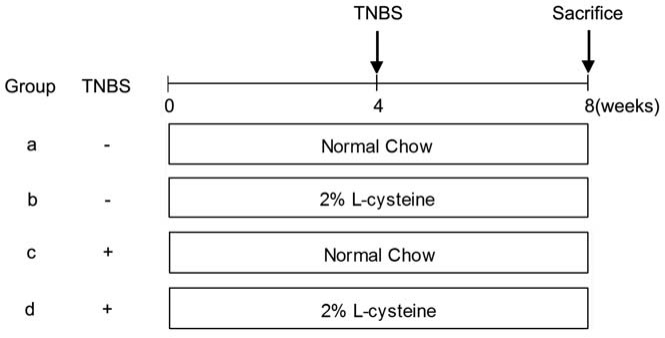
Schematic map of the experimental design. Four groups of rats (n = 10) were studied. Groups a and b received saline injections (no induction of chronic pancreatitis), groups c and d received TNBS injections (0.4 ml of 2% TNBS for the induction of chronic pancreatitis). Groups a and c received normal chow throughout the entire 8-week study period. Groups b and d received chow mixed with 2% L-cysteine during the 8-week study, after which the rats were sacrificed. Arrows indicate injections with TNBS.

One-month-old male Sprague Dawley (SD) rats (50 g–80 g) were purchased from Shanghai SLAC Laboratory Animal Co Ltd (Shanghai, China). Rats were randomly assigned to four groups of 10 animals each (Figure 1). In this study, group b and group d rats were fed with rat chow containing 2% L-cysteine (Sigma-Aldrich, St. Louis, Missouri, USA) for the duration of the experiment, according to a protocol established by Horie et al. [18], and group a and group c rats were given normal chow without L-cysteine. One month after the initiation of the diet, group a and group b rats were given sham operations, and group c and group d rats were induced in the experimental model of chronic pancreatitis as previously described [19]. Briefly, the main pancreatic duct of anesthetized rats, using 3% pentobarbital sodium, was cannulated through the papilla using polyethylene tubing (PE 5.0). The duct was tied close to the liver and 0.4 ml of 2% TNBS (Sigma-Aldrich, St. Louis, Missouri, USA) solution (in 10% ethanol in phosphate-buffered saline (PBS, PH 7.4)) was intraductally infused until completion. Total exposure time to TNBS was 40 minutes followed by a washout period of 30 minutes. Ligatures were then released and the duodenum and the abdominal wall were sutured. 28 days after TNBS injection, rats were killed under anesthesia with 3% pentobarbital sodium and each pancreas was quickly removed and weighed, fixed in 4% paraformaldehyde buffered with PBS overnight at 4°C, and embedded in paraffin wax or frozen immediately at −80°C. Whole-blood samples were kept at room temperature for 2 hours before centrifugation for 20 minutes at ∼2000×g, and serum was stored at −80°C for further studies. Dead rats were replaced with new ones to maintain 10 animals in each group.

### Serum amylase and lipase assay

The serum activities of amylase and lipase were measured by enzyme dynamics chemistry using commercial kits according to the manufacturer's protocols in a Roche/Hitachi modular analytics system (Roche, Mannheim, Germany).

### Histological examination

For light microscopy, haematoxylin-eosin (H&E) staining and Masson's trichrome staining were done according to standard procedures. All specimens were scored by 2 pathologists who were unaware of the origin of the specimens. Evaluation of the pancreas was performed according to Puig-Divi et al. [20]. Three pancreas sections were randomly selected and scored from each rat. Median scores were calculated to morphologically assess tissue damage. Briefly, a semi-quantitative score was used that graded glandular atrophy (0–3), intralobular, interlobular and periductal fibrosis (0–3) and inflammatory mononuclear infiltrates (0–3). A damage index (DI) was established.

### Immunohistochemical study of alpha-smooth muscle actin (α-SMA)

Tissue block sections were mounted on slides, deparaffinized in xylene and rehydrated in alcohol. Endogenous peroxidase was blocked with 3% hydrogen peroxide. Antigen retrieval was achieved by microwave using EDTA buffer (pH 9.0). Sections were then incubated overnight at 4°C with a monoclonal antibody mouse α-SMA (1∶800 dilution, Santa Cruz, California, USA). The antibody binding was detected with an Envision™ Detection Kit, Peroxidase/DAB, Rabbit/Mouse (Gene Tech, Shanghai, China). Then the sections were counterstained with haematoxylin. For negative control, the buffer replaced the primary antibody. The positive areas stained with α-SMA was examined in all specimens using a microscope (CTR 6000; Leica, Wetzlar, Germany) and analyzed by using image analysis software (Image Pro Plus software, Media Cybernetics, Gleichen, Germany).

### Immunofluorescence staining of α-SMA and collagen 1α1

Paraffin-embedded pancreas samples were deparaffinized and rehydrated. Sections were microwave treated (5×5 minutes) in EDTA buffer (pH 9.0), allowed to cool for 30 minutes, and washed in PBS (3×5 minutes). After being blocked for 20 minutes with 5% bovine serum albumin, slides were incubated overnight at 4°C with a mouse monoclonal antibody against α-SMA (1∶200 dilution) and rabbit polyclonal antibodies against collagen 1α1 (1∶50 dilution). Sections were then rinsed in PBST (PBS+0.1% Tween-20) and immunoreactive protein was detected using a donkey anti-mouse antibody (1∶400 dilution) conjugated with fluorochrome Cy3 (Jackson ImmunoResearch Laboratory, USA) and a donkey anti-rabbit antibody (1∶200 dilution) conjugated with fluorochrome Alexa Fluor® 488 (Jackson ImmunoResearch Laboratory, USA) for 1 h in the dark. After being rinsed in PBST, slides were mounted with Fluoromount™ mounting medium (Sigma-Aldrich, St. Louis, Missouri, USA) with 4′, 6-diamidino-2-phenylindole (DAPI) (1∶1000 dilution). Fluorescence analysis was performed by using a confocal laser scanning microscope (LSM 710; Zeiss, Germany) and Zen 2009 software (Carl-Zeiss, Jena, Germany).

### Hydroxyproline assay

Intrapancreatic hydroxyproline was quantified using the detection kit according to Reddy and Enwemeka [21] and the manufacturer's instructions (Jiancheng Bioengineering Institute, Nanjing, China). Hydroxyproline content is expressed as micrograms of hydroxyproline per gram pancreatic tissue.

### Cell culture and immunocytochemistry of bromodeoxyuridine (BrdU)

PSCs were isolated from pancreas by the method described by Apte et al [22]. Freshly isolated rat PSCs were seeded on 1-cm^2^ uncoated glass coverslips in 6-well plates (10 cm^2^/well; two to three glass coverslips per well) and cultivated in DMEM/F12 (Gibco BRL, USA) supplemented with 10% fetal bovine serum (FBS; Gibco BRL, USA) and 1% penicillin–streptomycin (Gibco BRL, USA) at 37°C, 5% CO_2_. On the next day, the culture medium was changed to MEM+0–10 mM L-cysteine. After a 3-day exposure to L-cysteine, some coverslips were treated with 10 µM Brdu (Sigma-Aldrich, St. Louis, Missouri, USA) and fixed in 4% paraformaldehyde. After 5-day exposure to L-cysteine, the other coverslips were fixed in 4% paraformaldehyde. Then cells were immunostained for Brdu and α-SMA essentially as described above for tissue sections.

### Effect of L-cysteine on acinar cells' viability

Acinar cells were isolated according to the procedure described by Hu et al [23]–[25]. Acinar cells (5×10^4^/well) were seeded in 24-well plates in DMEM/F12 supplemented with 10% FBS and 1% penicillin–streptomycin at 37°C, 5% CO_2_. 24 hours later, the cells were treated with MEM+0–10 mM L-cysteine and incubated for 3 days. Cell viability was determined by Cell Counting Kit-8 (Dojindo, Kumamoto, Japan) according to manufacturer's instruction.

### Cell cycle analysis by flow cytometry

When 30% confluence was achieved, PSCs were synchronized for 24 h with 0.4 µg/ml Demecolcine (Sigma-Aldrich, St. Louis, Missouri, USA) and treated with 0–1 mM L-cysteine, After 3 days, the cells were collected with trypsin, washed 3 times with PBS, and fixed in 70% ethanol at 4°C. The cells were then suspended in a solution containing Nonidet P-40 and ribonuclease A and, after staining with 0.5 mg/ml propidium iodine, S-phase fractions and cell-cycle kinetics were carried out using a FACS Calibur (Becton Dickinson,San Jose, CA) using CELL Quest version 3.3 software.

### Oxidative stress analysis

The levels of malondialdehyde (MDA) and 4-hydroxynonenal (4-HNE) in fresh pancreatic tissues and PSCs were measured to assess lipid peroxidation, using LPO-586 commercial kit (ENZO Life Sciences, Inc., Farmingdale, NY), according to the manufacturer's protocol. MDA+4-HNE levels were measured spectrophotometrically and were measured in triplicate.

Glutathione (GSH) concentrations were estimated in the pancreas samples and PSCs using Total Glutathione Quantification Kit (Dojindo Molecular Technologies Inc, Kunamoto, Japan) according to the manufacturer's protocol.

Protein concentration was determined by the standard BCA method (BCA™ Protein Assay Kit, Pierce, USA).

### Real Time Quantitative PCR (qRT-PCR)

Total RNA was isolated from pancreas of CP rats using TRIzol reagent (Invitrogen, Carlsbad, CA, USA) and then quantified. RT reactions were performed with total RNA (2 µg) according to the ExScript RT reagent kit. qRT-PCR was performed in triplicate for each gene of interest under each triplicate experimental condition using ABI Prism 7900HT Sequence Detection System (Applied Biosystems, CA, USA). GAPDH was used as separate endogenous controls to which each gene of interest was normalized. Fold changes and subsequent percent gene expression levels relative to designated control groups were calculated using the comparative CT (2^−ΔΔCT^) method. The primer sequences are as follows: Nuclear erythroid-related factor 2 (Nrf2) (109 bp), forward: 5′-GCTATTTTCCATTCCCGAGTTAC-3′, reverse: 5′-ATTGCTGTCCATCTCTGTCAG-3′. NAD(P)H: quinine oxidoreductase-1 (NQO1) (197 bp), forward: 5′-CATCATTTGGGCAAGTCC-3′, reverse: 5′-ACAGCCGTGGCAGAACTA-3′. Heme oxidase-1 (HO-1) (102 bp), forward: 5′-CTTTCAGAAGGGTCAGGTGTC-3′, reverse: 5′-TGCTTGTTTCGCTCTATCTCC-3′. IL-1β (131 bp), forward: 5′-TGTGATGTTCCCATTAGAC-3′, reverse: 5′-AATACCACTTGTTGGCTTA-3′. GAPDH (140 bp) forward: 5′-TATCGGACGCCTGGTTAC-3′, reverse: 5′-CTGTGCCGTTGAACTTGC-3′.

### Western blot analysis

For western blot analysis, murine pancreas was rapidly ground in liquid nitrogen. The resulting powder was reconstituted in ice-cold RIPA buffer containing 1 mM phenylmethanesulfonyl fluoride(PMSF) and a cocktail of protease inhibitors (1∶100 dilution; Sigma-Aldrich). Primarily isolated PSCs treated with 0 mM-10 mM L-cysteine for 5 days were rinsed twice in PBS, then lysed for 2 h in RIPA lysis buffer on ice. After centrifuging the homogenates at 20,000 g for 10 minutes at 4°C, protein concentrations were determined. Equal amounts of protein (20 µg from cells or 40 µg from tissues) were electrophoresed through sodium dodecyl sulfate/polyacrylamide gels (Bio-Rad, California, USA) and transferred electrophoretically to membranes. Nonspecific binding was blocked by 1 h incubation of the membrane in 5% low-fat milk. The blots were then incubated with a primary antibody overnight at 4°C. Following incubation with peroxidase-conjugated secondary antibodies, proteins were visualized using the ECL-detection system (Santa Cruz Biotechnology, Santa Cruz, CA), quickly dried, and exposed to ECL film.

Primary antibodies were as follows: anti-α-SMA (1∶250 dilution; Santa Cruz Biotechnology, Santa Cruz, CA), anti-transforming growth factor-β1 (TGF-β1) (1∶250 dilution; Santa Cruz Biotechnology, Santa Cruz, CA), anti-collagen 1α1 (1∶250 dilution; Santa Cruz Biotechnology), anti-GAPDH (1∶1,000 dilution; Epitomics), anti-tissue inhibitors of metalloproteinase 1 (TIMP1) (1∶100 dilution; Santa Cruz Biotechnology), anti-matrix metalloproteinase 2 (MMP2) (1∶100 dilution; Santa Cruz Biotechnology), anti-interleukin-1β (IL-1β) (1∶200 dilution; Santa Cruz Biotechnology), anti-PDGFRβ (1∶200 dilution; Cell Signaling Technology;Danvers, MA, USA) and anti-TGFβRII (1∶200 dilution; Santa Cruz Biotechnology, Santa Cruz, CA).

### Statistical analysis

Data are presented as the mean±standard deviation (SD). Statistical analysis was performed using one-way analysis of variance (ANOVA) followed by SNK tests as a *post hoc* test. The Kruskal-Wallis test was used to evaluate the differences in categorical values followed by Mann-Whitney U tests as a *post hoc* test. p value of <0.05 was accepted as statistically significant.

## Results

### Effects of L-cysteine on serum indexes, body weight

L-cysteine showed no effects on serum amylase, lipase and body weight changes both in control groups and TNBS treated groups (Figures S1, S2).

### L-cysteine had no side effect on important organs and on viability of primarily isolated acinar cells

In vitro study showed that there was no obvious morphological difference between acinar cells treated with or without L-cystein for 3 days (Fig. S3A, S3B) and the viability of acinar cells was not affected by L-cysteine administration (Fig. S3C).

In addition, *in vivo* study showed that there were not obvious histological changes in lung, liver, intestine, kidney between group c and group d (Fig. S4).

### L-cysteine attenuated chronic pancreatitis in rats induced by TNBS

**Figure 2:**
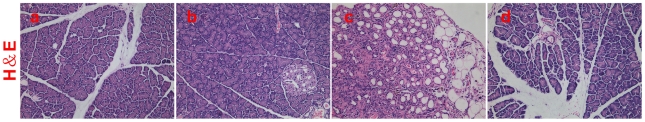
Histological observations in H&E stained sections. For the description of groups a to d, see Figure 1. Abnormal architecture, glandular atrophy, pseudotubular complexes, fibrosis and inflammatory cell infiltration can be seen in group c, while appearance is fairly normal in groups a and b. Representative H&E sections of rats revealed that 2% L-cysteine attenuated the development of pancreatic fibrosis induced by TNBS (group d). Representative original magnification ×200.

Two months feeding of L-cysteine showed no significant histological changes between sham groups (groups a and b), indicating a long term treatment with L-cysteine was not toxic to the normal pancreas by tissue section observations. In TNBS treated rats (groups c and d), there were histopathological signs of chronic pancreatitis at the time of sacrifice (week 8), as reflected by abnormal architecture, glandular atrophy, pseudotubular complexes, fibrosis, and inflammatory cell infiltrates (Table 1 and Figure 2; all p*<*0.05 for the comparisons with groups a and b). L-cysteine administration (group d) led to less severe pancreatic damage in CP rats in terms of all the evaluating scores mentioned above (Table 1 and Figure 2; all p<0.05 for the comparisons with group c).

**Table 1:** L-cysteine reduces the severity of experimental chronic pancreatitis.

	a	b	c	d
**Glandular atrophy**	0±0	0±0	2.10±0.18^#^	1.50±0.16^#^
**Fibrosis**	0±0	0±0	2.50±0.17^#^	1.20±0.20^**#^
**Inflammation**	0±0	0±0	2.30±0.21^#^	1.40±0.22^*#^
**Damage index(DI)**	0±0	0±0	6.90±0.35^#^	4.10±0.35^**#^

For the description of the histopathological score, see the Materials and Methods section. Data were expressed as mean±SD (n = 10). A Mann-Whitney *U* test was used to evaluate the differences among the groups. Chronic pancreatitis groups showed pancreatic fibrosis and damage (^#^p<0.01 *vs*. groups a and b). L-cysteine administration alleviated pancreatic fibrosis (*p<0.05 *vs*. group c, **p<0.01 *vs*. group c).

### L-cysteine prevented fibrosis in pancreatic tissue

**Figure 3:**
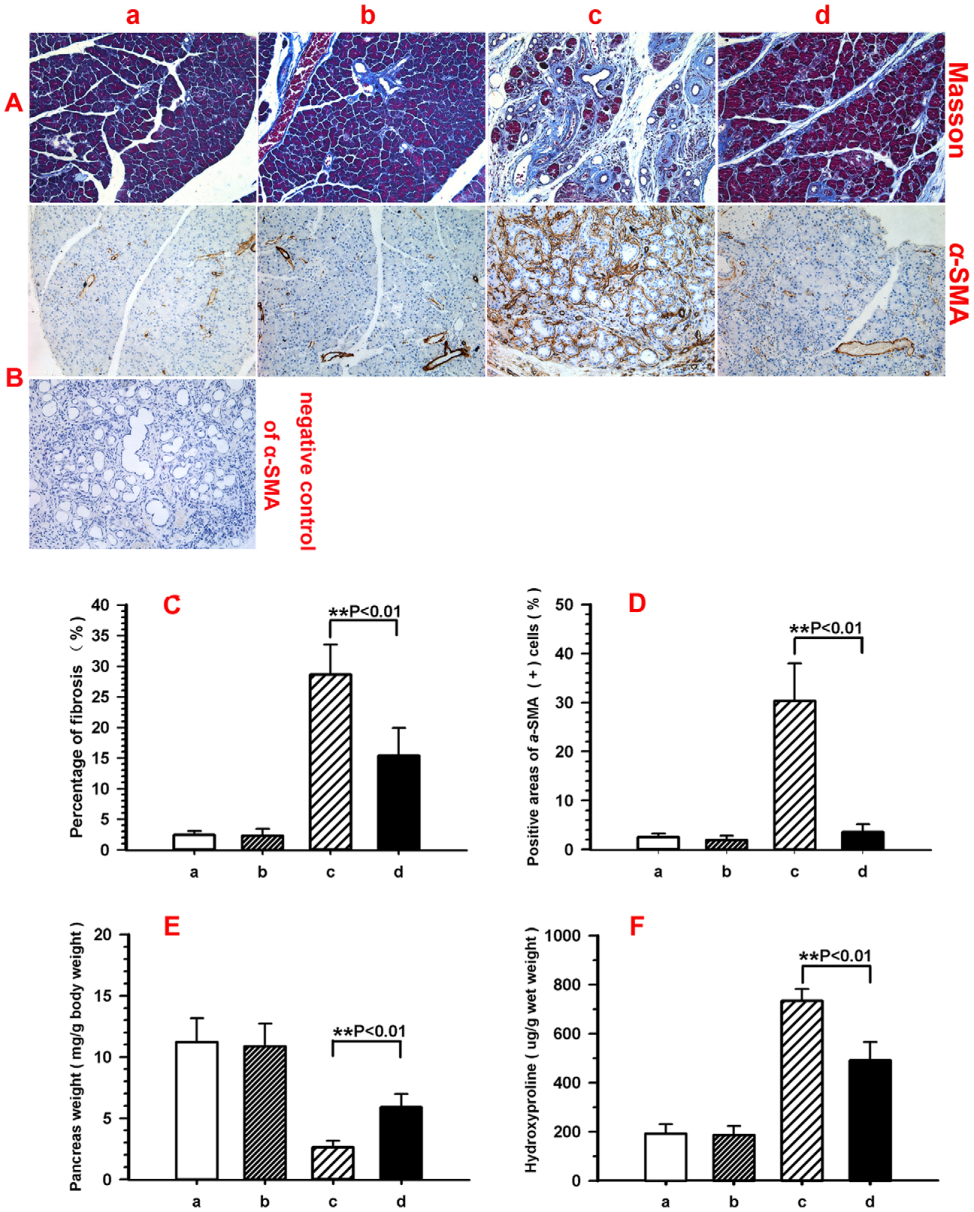
L-cysteine attenuates pancreatic fibrosis induced by TNBS. For the description of groups a to d, see Figure 1. (A, B) Pancreatic tissue sections stained with Masson and stained immunohistochemically for α-SMA. (C, D) Quantification of the positive areas of Masson and α-SMA by Image Pro Plus. (E) Effect of L-cysteine treatment on pancreas wet weight. Pancreas wet weight was significantly increased in L-cysteine treated rats. (F) Effect of L-cysteine treatment on pancreatic content of hydroxyproline after TNBS injury. Values are mean±SD (n = 10). Significant differences: **p<0.01 group c *vs*. group d. Representative original magnification ×200.

To evaluate the degree of fibrosis in the pancreas, Masson-stained pancreas sections were analyzed using computer-assisted digital image analysis. Pancreatic collagen content dramatically increased after induction of CP (Figure 3A–C). However, the increase in pancreatic collagen content was diminished by L-cysteine feeding (Figure 3A–D). Positive areas of Masson-stained sections of group c were higher compared with group a (Figure 3C, 2.41±0.66 *vs.* 28.63±4.91%; p<0.01 group a *vs.* group c). 2.0% L-cysteine diet (group d) decreased the positive areas of Masson-stained sections compared with group c (Figure 3C, 28.63±4.91% *vs.* 15.36±4.55%; p<0.01 group c *vs.* group d).

Immunohistochemistry (IHC) of α-SMA was performed to quantify the number of activated stellate cells, the expression of which was obviously increased during TNBS-induced pancreatitis and reduced by L-cysteine treatment (Figure 3B). Positive areas of α-SMA-stained sections were higher in group c than that in group a (Figure 3D, 30.31±7.69% *vs*. 2.55±0.69%; p<0.01). A 2.0% L-cysteine diet (group d) decreased the positive areas of α-SMA-stained sections (Figure 3D, 30.31±7.69% *vs*. 3.52±1.65%; p<0.01).

A marked decrease in pancreas wet weight secondary to pancreatic atrophy after chronic injury, was noted in group c (Figure 3E, 2.64±0.51 mg/g body weight) compare with group a (Figure 3E, 11.21±1.96 mg/g body weight) and b (10.86±0.59 mg/g body weight) and were significantly attenuated in group d (Figure 3E, 5.90±.07 mg/g body weight), which included supplementation with 2% L-cysteine.

For further confirmation of the anti-fibrotic role of L-cysteine in CP rats, the content of hydroxyproline in the pancreas was also assayed, which was significantly increased to (732.84±48.93) µg/g wet weight in group c on day 28 compared with group a (191.50±38.25 µg/g wet weight) and group b (185.85±36.66 µg/g wet weight), whereas in group d, it decreased to (490.15±75.79) µg/g wet weight (Figure 3F), showing a tendency of reduction by L-cysteine in the accumulation of collagens in the pancreas of CP rats.

**Figure 4:**
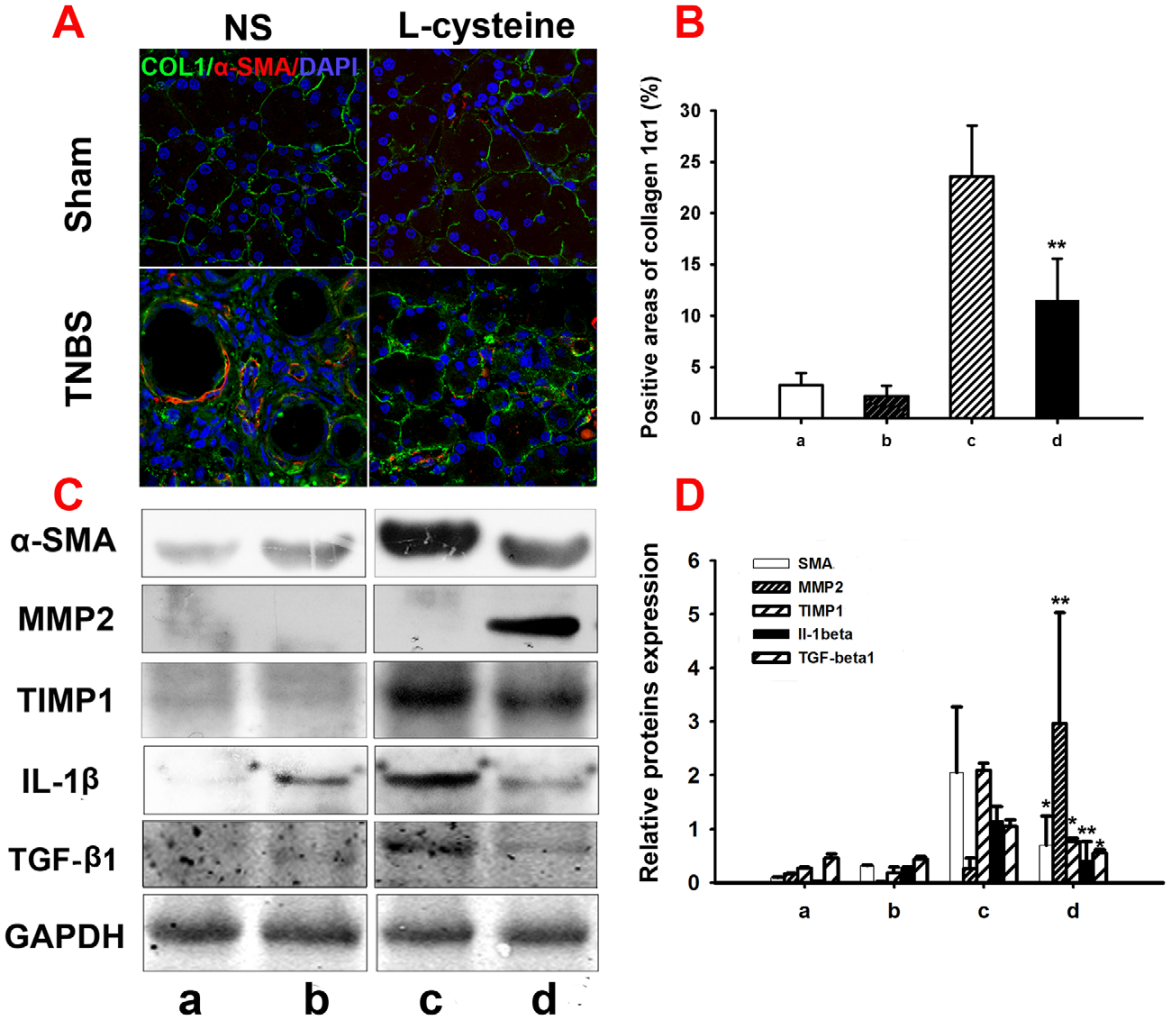
L-cysteine modulates extracellular matrix secretion in vitro. For the description of groups a to d, see Figure 1. (A, B) Double immunofluorescence of collagen 1α1 (green) and α-SMA (red) in the pancreas, 4′, 6-Diamidino-2-phenylindole (DAPI; blue) was used to counterstain nuclei. The co-localization of collagen 1α1 and α-SMA is highlighted by the yellow color. Immunostainning showed a low expression of collagen 1α1 in the sham pancreas, but its expression increased obviously after 4 weeks TNBS treatment, while L-cysteine administration attenuated collagen 1α1 expression in CP rats. (C, D) Expression of α-SMA, MMP2, TIMP1, TGF-β1 and IL-1β proteins in 4 groups of pancreatic tissues were detected by western blot analysis. GAPDH was used as the loading control in all experiments. The results were quantified by determining the intensities of the bands compared with those of GAPDH. All data are presented as the mean±SD of three independent experiments. Significant differences: *p<0.05 *vs*. group c, **p<0.01 *vs*. group c. Representative original magnification ×400.

To confirm the presence of PSCs in fibrotic areas immunofluorescence staining of α-SMA and collagen 1α1 was conducted (Figure 4A). In normal pancreas collagen 1α1 appeared as loose fibres surrounding acinar units and pancreatic islets. Intense immunostaining for a-SMA was found in cells of vessels located within interlobular septa, while cells of pancreatic lobules showed no staining. In CP rats intense immunostaining for collagen 1α1 was present in the fibrous septa and in the fibrotic stroma surrounding pancreatic acini. The deposition of collagen 1α1 was markedly enhanced in comparison to normal tissues. Meanwhile the number of cells expressing a-SMA was increased in the fibrotic tissue, particularly in the connective tissue surrounding fibrotic acini and at the interface between fibrotic septa and lobules. L-cysteine administration led to less expression of a-SMA and collagen 1α1. A same tendency was shown in the analysis for positive areas of collagen 1α1-stained sections (Figure 4B).

Along with the IHC assay, the expression of collagen deposition related proteins and cytokines, including α-SMA, TIMP1, TGF-β1 and IL-1β, increased significantly after TNBS treatment compared with normal rats and decreased obviously after L-cysteine administration as shown in Figure 4C and 4D. An opposite tendency was observed in the expression of MMP2.

### L-cysteine inhibited the proliferation and activation of PSCs in vitro

**Figure 5:**
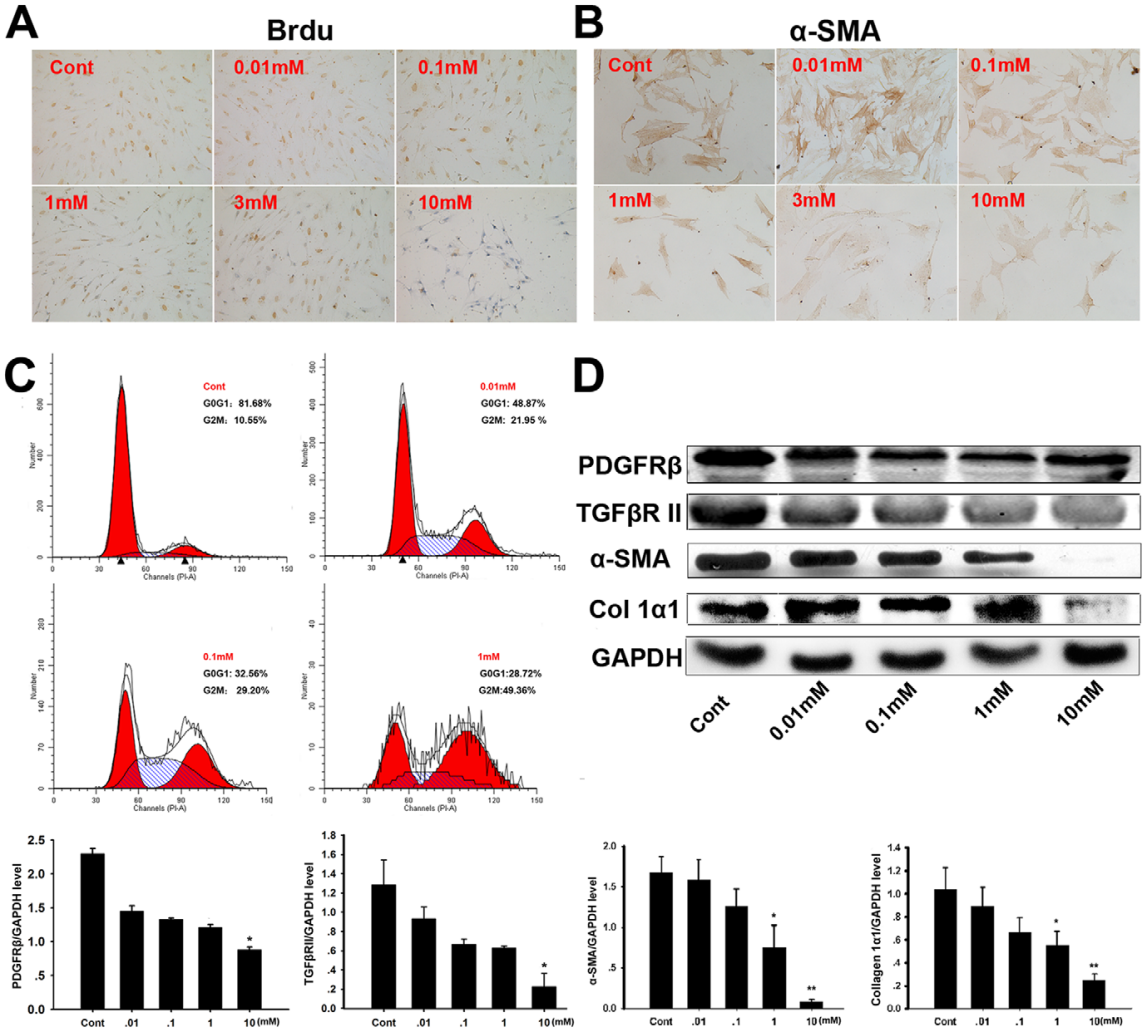
L-cysteine inhibits the proliferation and activation of PSCs. (A) Brdu staining of PSCs three days after L-cysteine treatment, DNA synthesis was detected by BrdU incorporation during the final 2 hours (dividing cells are stained dark brown). (B) Effect of L-cysteine on the activation of freshly isolated PSCs. After culturing PSCs isolated from a rat for 24 hour, the medium was changed to MEM+0–10 mM L-cysteine. PSCs were cultured for five days under the above conditions and α-SMA expression by PSCs was measured with immunocytochemistry. (C) The cell cycle distribution of PSCs at different concentrations of L-cysteine was analyzed by flow cytometry. (D) Expression of PDGFRβ, TGFβRII, α-SMA and collagen 1α1 protein in the PSCs treated with different concentrations of L-cysteine was analyzed by western blot. GAPDH was used as a loading control. The intensities of bands were measured, and the represented values correspond to the PDGFRβ, TGFβRII, α-SMA and collagen 1α1/GAPDH ratio. All data are presented as the mean±SD of three independent experiments. Significant differences: *p<0.05 *vs*. control group, **p<0.01 *vs*. control group. Representative original magnification ×200.

To evaluate whether L-cysteine affected the proliferation of activated PSCs *in vitro*, BrdU incorporation into the nucleus of PSCs was monitored and the inhibition of DNA synthesis by L-cysteine showed a concentration-dependent manner (Figure 5A). Inhibition of BrdU incorporation was first seen at 1 mM L-cysteine. There was a significant reduction in proliferation rates when the culture media was supplemented with 10 mM L-cysteine. α-SMA was characterized as a marker of PSC activation and its expression was decreased by L-cysteine, and it was especially inhibited by 10 mM L-cysteine (Figure 5B). The cell cycle distribution was analyzed by flow cytometry. Compared with 10% FBS alone, L-cysteine decreased the percentage of cells in G0/G1 and increased the percentage of cells in G2/M phase in a concentration-dependent manner (Figure 5C). Western blot analysis revealed that the expression of PDGFRβ, TGFβRII, collagen 1α1 and α-SMA in PSCs was suppressed by supplementation with L-cysteine, especially at 10 mM (Figure 5D).

### Oxidative stress analysis

**Figure 6:**
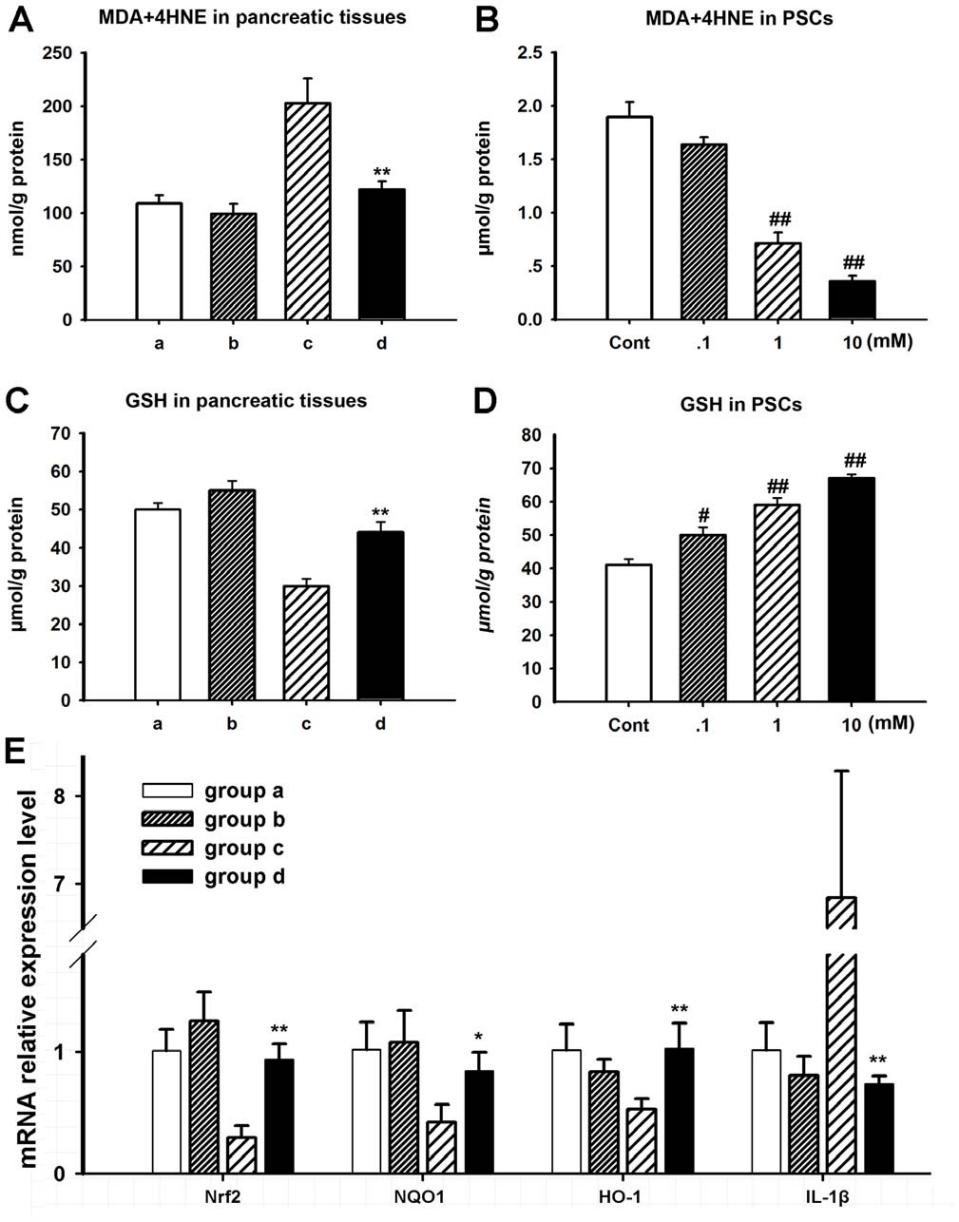
L-cysteine modulates oxidative stress *in vitro* and *in vivo* and regulates Nrf2 associated pathway. For the description of groups a to d, see Figure 1. (A) MDA+4HNE concentration of pancreatic tissues. MDA+4HNE concentration of pancreatic tissues increased significantly after induction of CP compared with sham groups and decreased obviously after L-cysteine administration. (C) GSH levels of pancreatic tissues. GSH levels of pancreatic tissues decrease significantly after TNBS administration compared with sham groups and increased obviously after L-cysteine administration. (B, D) In vitro study, L-cysteine treatment affected MDA+4-HNE and GSH levels in a dose-dependent manner in PSCs as that in *in vivo* study. (E) Relative mRNA levels of Nrf2, NQO1, HO-1 and IL-1β after L-cysteine treatment in pancreas of four groups. All data are presented as the mean±SD of three independent experiments. Significant differences: *p<0.05 *vs*. group c, **p<0.01 *vs*. group c, # p<0.05 *vs*. control group, ## p<0.01 *vs*. control group.

MDA+4-HNE levels and GSH concentrations in pancreatic tissues and PSCs were analyzed to determine the oxidative stress status (Figure 6). The contents of MDA+4-HNE in pancreatic tissues were found significantly increased after TNBS treatment compared with sham group, but decreased obviously after L-cysteine administration (p<0.01 group c *vs.* group d). The total GSH levels in pancreatic tissues were found lower in TNBS treated group compared with the sham control group and L-cysteine treatment significantly increased the GSH levels compared with those in group c (p<0.01 group c *vs.* group d). Similarly, a dose-dependent change of the levels of MDA+4-HNE and GSH could be seen in PSCs in vitro after L-cysteine treatment.

### Effect of L-cysteine on Nrf2 expression and its regulated genes in pancreas of CP rats

To determine whether Nrf2 and Nrf2 regulated genes could be induced by L-cysteine in CP rats, mRNA expression of Nrf2, NQO1, HO-1, and IL-1β was determined by qRT-PCR. mRNA expression of Nrf2 in rats of group c was low compared with it of sham groups (Fig. 6E). However, its mRNA expression increased after L-cysteine administration (P<0.01). In addition, mRNA expression of NQO1 and HO-1 in rats of group d was similarly significantly increased compared with group c while IL-1β had an opposite tendency.

## Discussion

Girish and colleagues observed significant reduction in plasma sulphur containing amino acids, such as methionine and cystine, in patients with chronic pancreatitis, suggesting the potential role of these amino acids in the pathogenesis of this disease [26]. Horie et al. showed that L-cysteine is effectively against liver fibrosis. The mechanism of inhibition of fibrosis in the liver was suggested to be the direct inhibition of activated Hepatic stellate cell (HSC) proliferation and HSC transformation by L-cysteine [18]. Matsui et al. found that L-cysteine and L-methionine regulated the activation of HSCs. Their oral intake aided suppression of the progression of liver fibrosis [27]. As PSCs share homologies with HSCs [28], our current results demonstrated that L-cysteine treatment also attenuated pancreatic fibrosis in CP rats.

The TNBS model exhibited morphological changes mimicking features of chronic pancreatitis in humans in TNBS treated rats [19], [20]. In our study, the pancreatic content of hydroxyproline was reduced by L-cysteine treatment and, histologically, H&E, Masson and α-SMA staining showed less pancreatic fibrosis in the L-cysteine treated group. Also, we demonstrated marked changes of the collagen deposition correlative protein. These findings indicated that pancreatic fibrosis induced by TNBS may be attenuated by L-cysteine administration.

In this model, PSCs activation was associated with fibrosis and activated PSCs were the main cellular source of collagen in CP [9]. In our study, an L-cysteine diet decreased the positive areas of α-SMA-stained sections compared with group treated with normal chow. This finding showed that L-cysteine may inhibit pancreatic fibrosis through inhibiting activation of PSCs. Analysis using primary-cultured PSCs revealed that this amino acid attenuated PSCs activation and proliferation, and the expression of PDGFRβ, TGFβRII, collagen 1α1 and α-SMA. This result is in line with Hiroko Matsui's result [27]. Reports showed that the proliferation of PSCs is associated with PDGF-ERK pathway, whereas the extracellular cell matrix(ECM) production of PSCs is associated with TGF-β related pathway [29]–[33]. Hence, the implication is that the beneficial effects of L-cysteine administration may be produced mainly by suppressing the proliferation and ECM production of PSCs through down-regulating the receptors of the two pathways mentioned above.

Molecular mechanisms for L-cysteine inhibiting fibrosis *in vivo* may be derived from its potential as a reducing agent in body metabolism. L-cysteine is utilized in the synthesis of proteins, non-protein compounds including taurine, reduced inorganic sulfur, sulfate, and GSH [15]. The major factors regulating GSH biosynthesis are the availability of cysteine [34]–[36]. Oxidative stress has been demonstrated to play an important role in the pathogenesis and progression of pancreatitis and also contributes to the development of pancreatic fibrosis [37]–[44]. Our current study showed that L-cysteine reduced the levels of MDA+4-HNE and enhanced the content of GSH in pancreatic tissues and PSCs, indicating its antioxidant effect which would ameliorate pancreatic fibrosis.

Another plausible reason is that L-cysteine may inhibit pancreatic fibrosis through modulating ECM deposition. A distorted balance of extracellular matrix synthesis and degradation in chronic pancreatitis is reported by several papers [1], [32], [45]. As mentioned before, PSCs have been shown to modulate the balance of extracellular matrix secretion and digestion by producing matrix metalloproteinases (MMPs) and their corresponding inhibitors, tissue inhibitors of metalloproteinases (TIMPs) [46]. In our study we observed an increase of MMP2 levels and a decrease of TIMP1 levels after treatment with L-cysteine which would reduce collagen synthesis. These findings show that L-cysteine may inhibit pancreatic fibrosis through modulating ECM deposition.

In addition, western blot analysis indicated that L-cysteine significantly down-regulated expression of TGF-β and IL-1βin CP rats, both of which are pivotal profibrotic cytokines as confirmed by several reports [13], [29], [47], [48]. By Stimulating the synthesis and secretion of collagens of PSCs, TGF-β promotes collagen deposition [29], [49], [50]. IL-1β has also been shown to have an important role in the pathogenesis of pancreatitis [48], [51]. IL-1β is a well described activator of pancreatic stellate cells [47]. Both TGF-β receptor knockout mice and IL-1β over-expression mice consistently develop severe chronic pancreatitis [51], [52]. Our results suggested that L-cysteine may inhibit pancreatic fibrosis through modulating inflammatory cytokine.

Recently, Nrf2 has emerged as an indispensable regulator of the constitutively inducible cytoprotective genes in various tissues and cell types [53]–[55]. In response to oxidative stress, Nrf2 accumulates in the nucleus, where it binds to Antioxidant Response Element (ARE) sequences in the regulatory sequences of its target genes which encode antioxidant enzymes and detoxifying proteins [56], [57]. For further investigation into the mechanism by which L-cysteine enhances antioxidant activity we observed the effect of L-cysteine on expression of Nrf2 and its downstream phase II antioxidant enzyme genes NQO1 and HO-1. Our study demonstrated that L-cysteine increased the mRNA expression of Nrf2 and its downstream genes NQO1 and HO-1 which enhance antioxidant defense capacity. Also, L-cysteine decreased the mRNA expression of IL-1β which in line with some reports using Nrf2 knock-out mice that showed increased expression of the inflammatory cytokine IL-1β compared with wide type mice [58], [59]. Thus, the results suggested that the increase of Nrf2 may be a plausible mechanism by which L-cysteine enhances antioxidant effect and modulates inflammatory cytokine.

It is worthwhile mentioning that L-cysteine was administered prior to the induction of CP, which does not simulate a clinical situation. Therefore, experiments should be established to study whether L-cysteine is even beneficial in a therapeutic setting when given after initial damage.

In summary, L-cysteine has an anti-fibrotic effect on chronic pancreatitis induced by TNBS through inhibiting the activation and proliferation of PSCs and may be served as therapeutic potential agent for the treatment of pancreatic fibrosis. With PSCs as a star on the rise in pancreatic disease research [60], L-cysteine may also be considered as a potential inhibitor of PSCs in the future.

## Supporting Information

Figure S1 **Body weight of the CP rats.** We weighed the body weight of the rats in the 4 groups during the entire study to observe the effect of L-cysteine on nutrition. There were no obvious changes among the four groups.
(TIF)

Figure S2 **Serum amylase and lipase activity.** Serum amylase and lipase activity were measured in the 4 groups described above 4 weeks after TNBS injection. There were no significant changes among the four groups.
(TIF)

Figure S3 **Influence of different dose of L-cysteine on viability of acinar cells.** Primary isolated acinar cells cultured in DMEM/F12+10%FBS (×200). (B) Acinar cells cultured with L-cysteine for 3 days (at 10 mM, ×200). (C) Acinar cells were treated with increasing doses of L-cysteine for 3 days and cell viability was determined by CCK-8 kit.
(TIF)

Figure S4 **Histological observations in H&E stained sections of rat organs.** Representative H&E sections of rat lung (A), liver (B), intestine (C) and kidney (D) revealed that there are not obviously histological changes compared with group c after a long term administration of L-cysteine (group d). Representative original magnification ×200.
(TIF)

## References

[1] Apte MV, Wilson JS (2004). Mechanisms of pancreatic fibrosis.. Dig Dis.

[2] Clain JE, Pearson RK (1999). Diagnosis of chronic pancreatitis: is a gold standard necessary?. Surg Clin North Am.

[3] Khalid A, Whitcomb DC (2002). Conservative management of chronic pancreatitis.. Eur J Gastroenterol Hepatol.

[4] Laugier R, Grandval P (2002). Interventional treatment of chronic pancreatitis.. Eur J Gastroenterol Hepatol.

[5] Levy MJ, Wiersema MJ (2003). EUS-guided celiac plexus neurolysis and celiac plexus block.. Gastrointest Endosc.

[6] Freiss H, Berberat PO, Wirtz M, Buchler MW (2002). Surgical treatment and long-term follow up in chronic pancreatitis.. Gastroenterol Hepatol.

[7] Zhang W, Gao J, Zhao T, Wu W, Bai Y (2010). High-dose naproxen aggravates pancreatic fibrosis in a rat model of chronic pancreatitis.. Pancreas.

[8] Neuschwander-Tetri BA, Bridle KR, Wells LD, Marcu M, Ramm GA (2000). Repetitive acute pancreatic injury in the mouse induces procollagen alpha1(I) expression colocalized to pancreatic stellate cells.. Lab Invest.

[9] Haber PS, Keogh GW, Apte MV, Moran CS, Stewart NL (1999). Activation of pancreatic stellate cells in human and experimental pancreatic fibrosis.. Am J Pathol.

[10] Casini A, Galli A, Pignalosa P, Frulloni L, Grappone C (2000). Collagen type I synthesized by periacinar stellate cells (PSC) co-localizes with lipid peroxidationderived aldehydes in chronic alcoholic pancreatitis.. J Pathol.

[11] Ebert M, Kasper HU, Hernberg S, Friess H, Buchler MW (1998). Over expression of platelet derived growth factor (PDGF) B chain and type B PDGF receptor in human chronic pancreatitis.. Dig Dis Sci.

[12] Talukdar R, Tandon RK (2008). Pancreatic stellate cells: New target in the treatment of chronic pancreatitis.. Journal of Gastroenterology and Hepatology.

[13] Schneider E, Schmid-Kotsas A, Zhao J, Weidenbach H, Schmid RM (2001). Identification of mediators stimulating proliferation and matrix synthesis of rat pancreatic stellate cells.. Am J Physiol Cell Physiol.

[14] Kruse ML, Hildebrand PB, Timke C, Folsch UR, Schimdt WE (2000). TGF-β1 autocrine growth control in isolated pancreatic fibroblast cells/stellate cells in vitro.. Regul Pept.

[15] Martensson J, Bolin T (1986). Sulfur amino acid metabolism in chronic relapsing pancreatitis.. Am J Gastroenterol.

[16] Jesnowski R, Furst D, Ringel J, Chen Y, Schrodel A (2005). Immortalization of pancreatic stellate cells as an in vitro model of pancreatic fibrosis: deactivation is induced by matrigel and N-acetylcysteine.. Lab Invest.

[17] Apte MV, Phillips PA, Fahmy RG, Darby SJ, Rodgers SC (2000). Does alcohol directly stimulate pancreatic fibrogenesis? Studies with rat pancreatic stellate cells.. Gastroenterology.

[18] Horie T, Sakaida I, Yokoya F, Nakajo M, Sonakaa I (2003). L-cysteine administration prevents liver fibrosis by suppressing hepatic stellate cell proliferation and activation.. Biochemical and Biophysical Research Communications.

[19] Puig-Divi V, Molero X, Salas A, Guarner F, Guarner L (1996). Induction of chronic pancreatic disease by trinitrobenzene sulfonic acid infusion into rat pancreatic ducts.. Pancreas.

[20] Puig-Divi V, Molero X, Vaquero E, Salas A, Guarner F (1999). Ethanol feeding aggravates morphological and biochemical parameters in experimental chronic pancreatitis.. Digestion.

[21] Reddy GK, Enwemeka CS (1996). A simplified method for the analysis of hydroxyproline in biological tissues.. Clin Biochem.

[22] Apte MV, Haber PS, Applegate TL, Norton ID, McCaughan GW (1998). Periacinar stellate shaped cells in rat pancreas: identification, isolation, and culture.. Gut.

[23] Gukovskaya AS, Gukovsky I, Zaninovic V, Song M, Sandoval D (1997). Pancreatic acinar cells produce, release, and respond to tumor necrosis factor-alpha. Role in regulating cell death and pancreatitis.. J Clin Invest.

[24] Mareninova OA, Sung KF, Hong P, Lugea A, Pando SJ (2006). Cell death in pancreatitis: caspases protect from necrotizing pancreatitis.. J Biol Chem.

[25] Hu G, Shen J, Cheng L, Guo C, Xu X (2010). Reg4 protects against acinar cell necrosis in experimental pancreatitis.. Gut.

[26] Girish BN, Rajesh G, Vaidyanathan K, Balakrishnan V (2011). Alterations in plasma amino acid levels in chronic pancreatitis.. JOP.

[27] Matsui H, Ikeda K, Nakajima YJ, Horikawa S, Imanishi Y (2004). Sulfur-containing amino acids attenuate the development of liver fibrosis in rats through down-regulation of stellate cell activation.. Journal of Hepatology.

[28] Buchholz M, Kestler HA, Holzmann K, Ellenrieder V, Schneiderhan W (2005). Transcriptome analysis of human hepatic and pancreatic stellate cells: organ-specific variations of a common transcriptional phenotype.. J Mol Med.

[29] Apte MV, Haber PS, Darby SJ, Rodgers SC, McCaughan GW (1999). Pancreatic stellate cells are activated by proinflammatory cytokines: implications for pancreatic fibrogenesis.. Gut.

[30] Luttenberger TA, Schmid-Kotsas A, Menke A, Siech M, Beger H (2000). Platelet-derived growth factors stimulate proliferation and extracellular matrix synthesis of pancreatic stellate cells: implications in pathogenesis of pancreas fibrosis.. Lab Invest.

[31] Jaster R, Sparmann G, Emmirich J, Liebe S (2002). Extracellular signal regulated kinases are key mediators of mitogenic signals in rat pancreatic stellate cells.. Gut.

[32] Van Laethem JL, Robberecht P, Resibois A, Deviere J (1996). Transforming growth factor beta promotes development of fibrosis after repeated courses of acute pancreatitis in mice.. Gastroenterology.

[33] Yoo BM, Yeo M, Oh TY, Choi Jh, Kim WW (2005). Amelioration of pancreatic fibrosis in mice with defective TGF-beta signaling.. Pancreas.

[34] Kaplowitz N, Aw TY, Ookhtens M (1985). The regulation of hepatic glutathione.. Annu Rev Pharmacol Toxicol.

[35] Tateishi N, Higashi T, Shinya S, Naruse A, Sakamoto Y (1974). Studies on the regulation of glutathione level in rat liver.. J Biochem.

[36] Reed DJ, Orrenius S (1977). The role of methionine in glutathione biosynthesis by isolated hepatocytes.. Biochem Biophys Res Commun.

[37] Lemke M, Gorl N, Berg A, Weber H, Hennighausen G (2006). Influence of selenium treatment on the acute toxicity of dibutylin dichloride in rats.. Pancreatology.

[38] Ohashi S, ishio A, Nakamura H, Asada M, Tamaki K (2006). Overexpression of redox-active protein thioredoxin-1 prevents development of chronic pancreatitis in mice.. Antioxid Redox Signal.

[39] Tasci I, Deveci S, Isik AT, Comert B, Akay C (2007). Allopurinol in rat chronic pancreatitis: effects on pancreatic stellate cell activation.. Pancreas.

[40] Yan MX, Li YQ, Meng M, Ren HB, Kou Y (2006). Long-term high-fat diet induces pancreatic injuries via pancreatic microcirculatory disturbances and oxidative stress in rats with hyperlipidemia.. Biochem Biophys Res Commun.

[41] Zeki S, Miura S, Suzuki H, Watanabe N, Adachi M (2002). Xanthine oxidase Y derived oxygen radicals play significant roles in the development of chronic pancreatitis in wbn/kob rats.. J Gastroenterol Hepatol.

[42] Kirk GR, White JS, McKie L, Stevenson M, Young L (2006). Combined antioxidant therapy reduces pain and improves quality of life in chronic pancreatitis.. J Gastrointest Surg.

[43] Bhardwaj P, Garg PK, Maulik SK, Saraya A, Tandon RK (2009). A randomized controlled trial of antioxidant supplementation for pain relief in patients with chronic pancreatitis.. Gastroenterology.

[44] Yoo BM, Oh TY, Kim YB, Yeo M, Lee JS (2005). Novel antioxidant ameliorates the fibrosis and inflammation of cerulein-induced chronic pancreatitis in a mouse model.. Pancreatology.

[45] Shimizu K (2008). Pancreatic stellate cells: molecular mechanism of pancreatic fibrosis.. J Gastroenterol Hepatol.

[46] Phillips PA, McCarroll JA, Park S, Wu MJ, Pirola R (2003). Rat pancreatic stellate cells secrete matrix metalloproteinases: implications for extracellularmatrix turnover.. Gut.

[47] Mews P, Phillips P, Fahmy R, Korsten M, Pirola R (2002). Pancreatic stellate cells respond to inflammatory cytokines: potential role in chronic pancreatitis.. Gut.

[48] Bentrem DJ, Joehl RJ (2003). Pancreas: healing response in critical illness.. Crit Care Med.

[49] Vogelmann R, Ruf D, Wagner M, Adler G, Menke A (2001). Effects of fibrogenic mediators on the development of pancreatic fibrosis in a TGF-beta1 transgenic mouse model.. Am J Physiol Gastrointest Liver Physiol.

[50] Shek FW, Benyon RC, Walker FM, McCrudden PR, Pender SL (2002). Expression of transforming growth factor-beta 1 by pancreatic stellate cells and its implications for matrix secretion and turnover in chronic pancreatitis.. Am J Pathol.

[51] Bottinger EP, Jakubczak JL, Roberts IS, Mumy M, Hemmati P (1997). Expression of a dominant-negative mutant TGF-beta type II receptor in transgenic mice reveals essential roles for TGF-beta in regulation of growth and differentiation in the exocrine pancreas.. EMBO J.

[52] Marrache F, Tu SP, Bhagat G, Pendyala S, Osterreicher CH (2008). Overexpression of interleukin-1beta in the murine pancreas results in chronic pancreatitis.. Gastroenterology.

[53] Enomoto A, Itoh K, Nagayoshi E, Haruta J, Kimura T (2001). High sensitivity of Nrf2 knockout mice to acetaminophen hepatotoxicity associated with decreased expression of ARE-regulated drug metabolizing enzymes and antioxidant genes.. Toxicol Sci.

[54] Morimitsu Y, Nakagawa Y, Hayashi K, Fujii H, Kumagai T (2002). A sulforaphane analogue that potently activates the Nrf2-dependent detoxification pathway.. J Biol Chem.

[55] Lee JM, Li J, Johnson DA, Stein TD, Kraft AD (2005). Nrf2, a multi-organ protector?. FASEB J.

[56] Villeneuve NF, Lau A, Zhang DD (2010). Regulation of the Nrf2-Keap1 antioxidant response by the ubiquitin proteasome system: an insight into cullin-ring ubiquitin ligases.. Antioxid Redox Signal.

[57] Hayes JD, McMahon M, Chowdhry S, Dinkova-Kostova AT (2010). Cancer chemoprevention mechanisms mediated through the Keap1-Nrf2 pathway.. Antioxid Redox Signal.

[58] Khor TO, Huang MT, Kwon KH, Chan JY, Reddy BS (2006). Nrf2-deficient mice have an increased susceptibility to dextran sulfate sodium-induced colitis.. Cancer Res.

[59] Chowdhry S, Nazmy MH, Meakin PJ, Dinkova-Kostova AT, Walsh SV (2010). Loss of Nrf2 markedly exacerbates nonalcoholic steatohepatitis.. Free Radic Biol Med.

[60] Omary MB, Lugea A, Lowe AW, Pandol SJ (2007). The pancreatic stellate cell: a star on the rise in pancreatic diseases.. Journal of Clinical Investigation.

