# Supplementary figures and images for: L-Cysteine Administration Attenuates Pancreatic Fibrosis Induced by TNBS in Rats by Inhibiting the Activation of Pancreatic Stellate Cell
Source: PLoS One. 2012 Feb 16;7(2):e31807. doi: 10.1371/journal.pone.0031807 (PMC3281011; doi:10.1371/journal.pone.0031807)

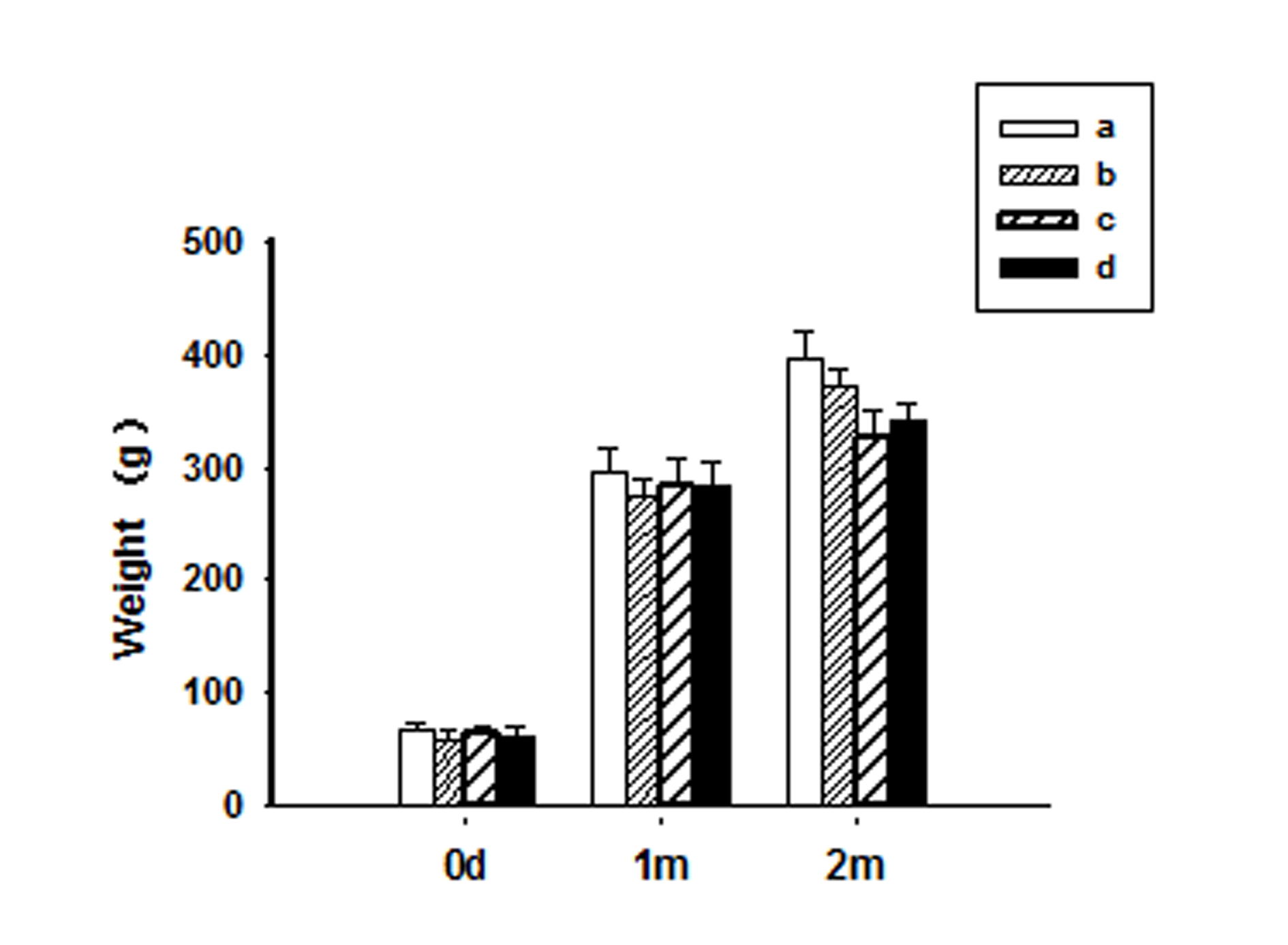

Supplement: Figure S1 — Body weight of the CP rats. We weighed the body weight of the rats in the 4 groups during the entire study to observe the effect of L-cysteine on nutrition. There were no obvious changes among the four groups. (TIF) [file pone.0031807.s001.tif]

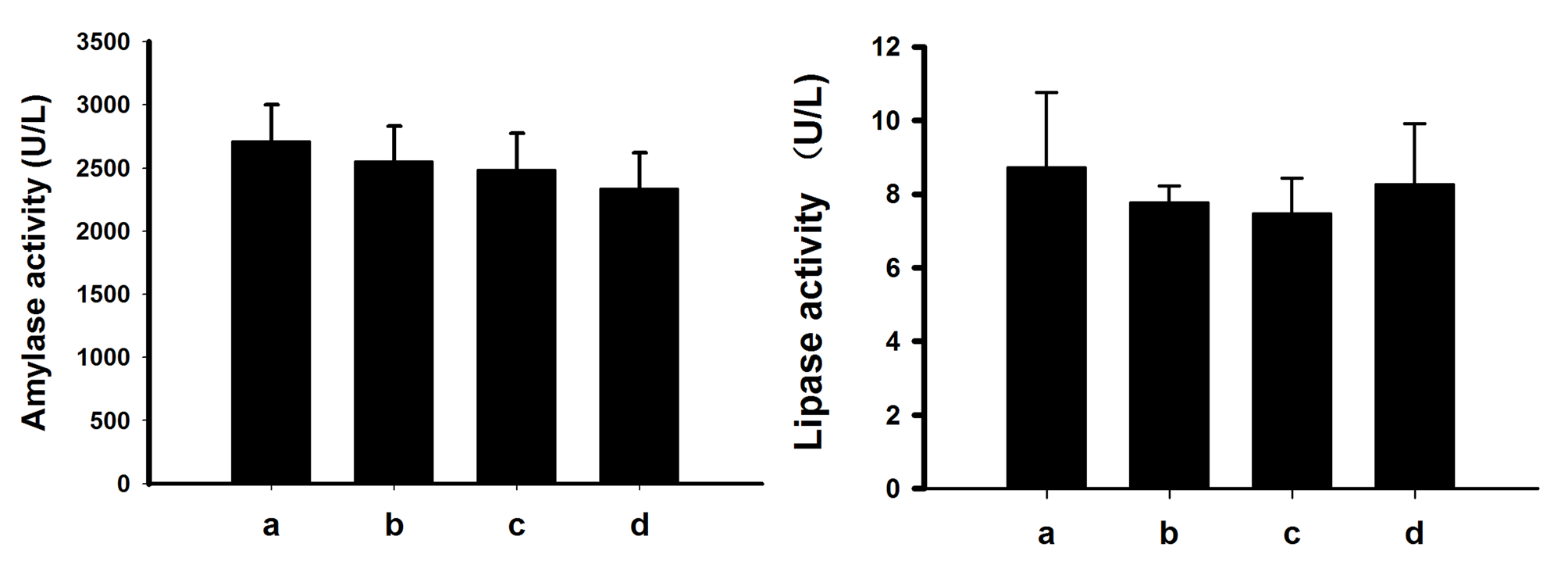

Supplement: Figure S2 — Serum amylase and lipase activity. Serum amylase and lipase activity were measured in the 4 groups described above 4 weeks after TNBS injection. There were no significant changes among the four groups. (TIF) [file pone.0031807.s002.tif]

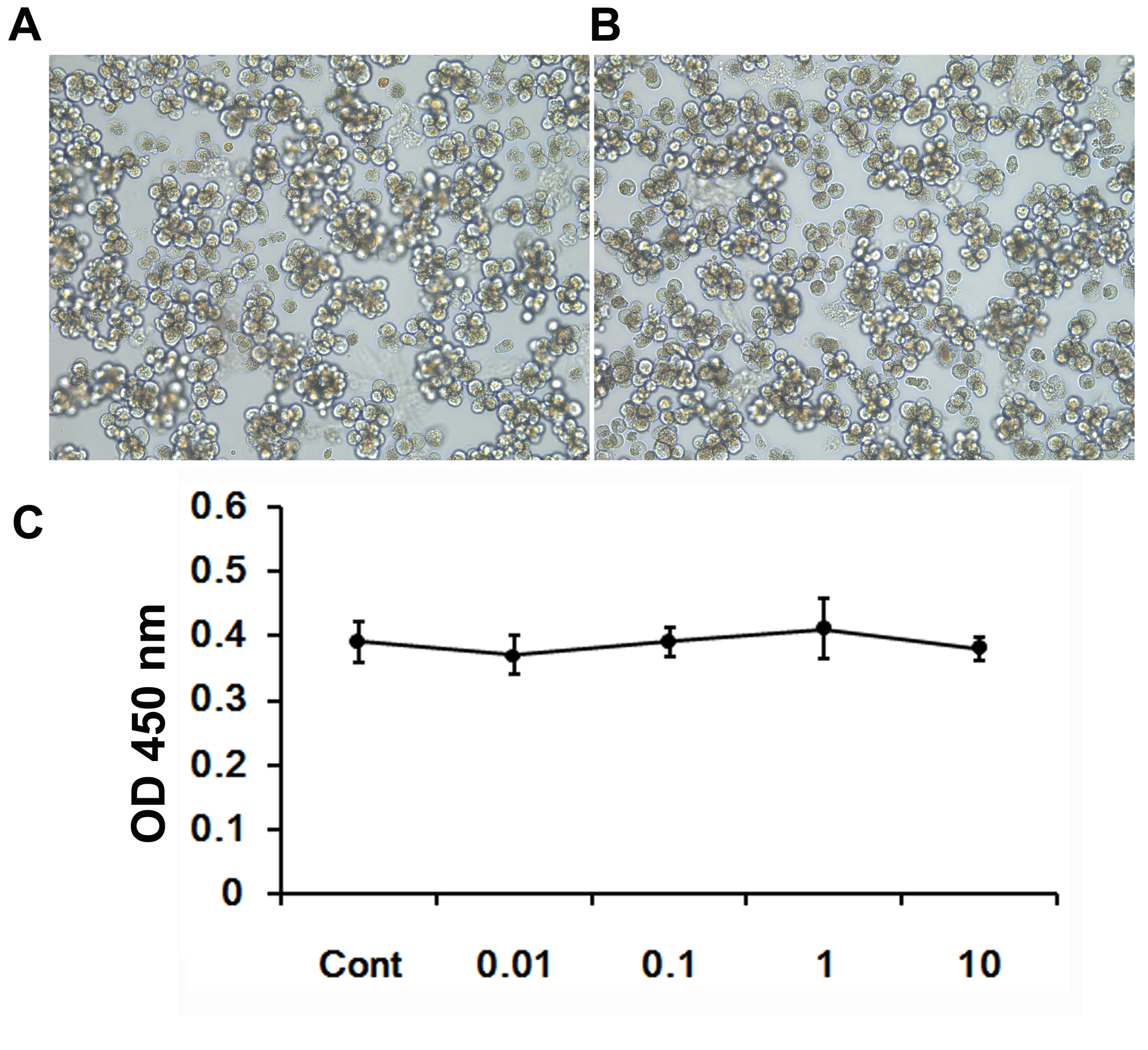

Supplement: Figure S3 — Influence of different dose of L-cysteine on viability of acinar cells. Primary isolated acinar cells cultured in DMEM/F12+10%FBS (×200). (B) Acinar cells cultured with L-cysteine for 3 days (at 10 mM, ×200). (C) Acinar cells were treated with increasing doses of L-cysteine for 3 days and cell viability was determined by CCK-8 kit. (TIF) [file pone.0031807.s003.tif]

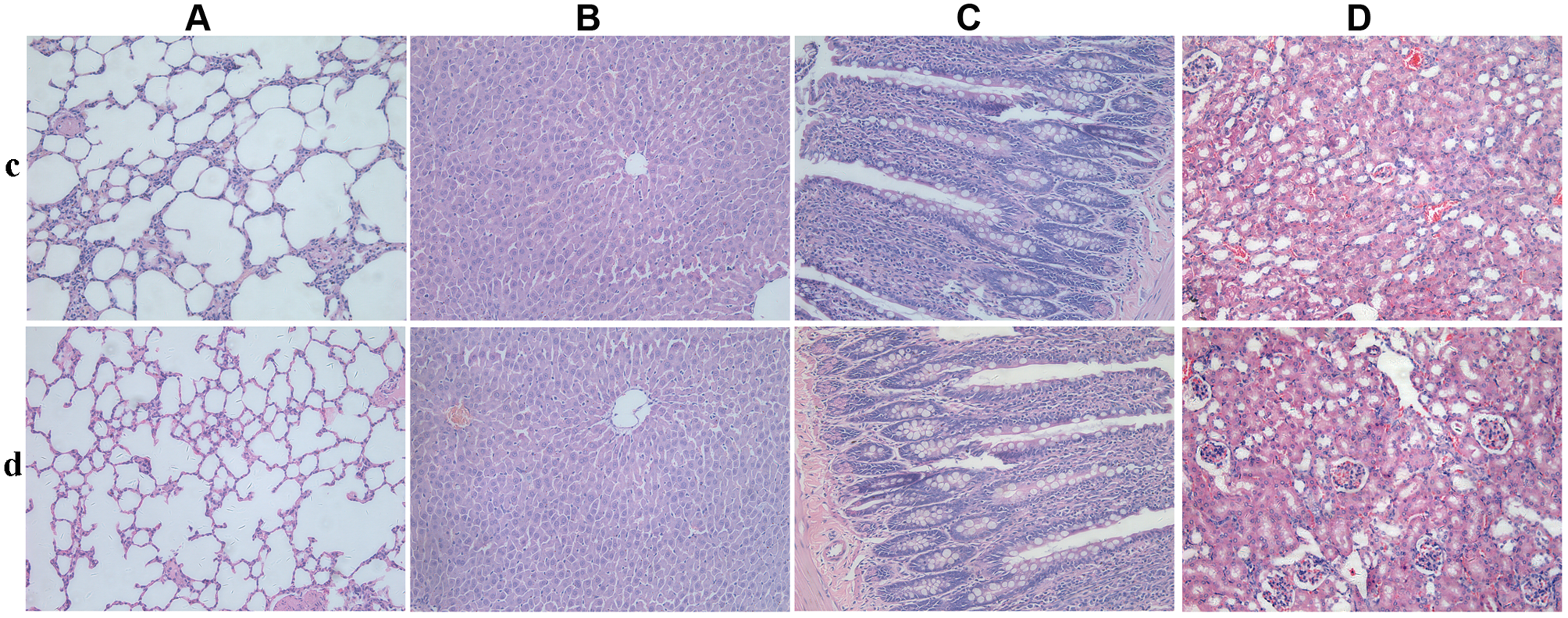

Supplement: Figure S4 — Histological observations in H&E stained sections of rat organs. Representative H&E sections of rat lung (A), liver (B), intestine (C) and kidney (D) revealed that there are not obviously histological changes compared with group c after a long term administration of L-cysteine (group d). Representative original magnification ×200. (TIF) [file pone.0031807.s004.tif]
